# Supplementary material for: Genetic variants associated with psychiatric disorders are enriched at epigenetically active sites in lymphoid cells
Source: Nat Commun. 2022 Oct 15;13:6102. doi: 10.1038/s41467-022-33885-7 (PMC9569335; doi:10.1038/s41467-022-33885-7)
Supplement: Supplementary file 1 — Supplementary Information [file 41467_2022_33885_MOESM1_ESM.pdf]

Genetic variants associated with psychiatric disorders are enriched at epigenetically active sites in lymphoid cells

Supplementary Material

Mary-Ellen Lynall[1,2,3,4,*], Blagoje Soskic[5,6,7], James Hayhurst[6], Jeremy Schwartzentruber[5], Daniel F. Levey[8,9], Gita A. Pathak[8,9], Renato Polimanti[8,9], Joel Gelernter[8,9,10], Murray B. Stein[11,12], Gosia Trynka[5,6], Menna R. Clatworthy[3,4], Ed Bullmore[1,2]

[1]University of Cambridge, Department of Psychiatry, Herchel Smith Building of Brain & Mind Sciences, Cambridge Biomedical Campus, Cambridge CB2 0SZ, UK

[2]Cambridgeshire & Peterborough NHS Foundation Trust, Cambridge, UK

[3]Molecular Immunity Unit, University of Cambridge Department of Medicine, Cambridge, UK

[4]Cellular Genetics, Wellcome Sanger Institute, UK

[5]Wellcome Sanger Institute, Wellcome Genome Campus, Cambridge, UK

[6]Open Targets, Wellcome Genome Campus, Hinxton, UK

[7]Human Technopole, Milan, Italy

[8]VA Connecticut Healthcare System, West Haven, CT, USA

[9]Yale Univ. School of Medicine, Dept Psychiatry, New Haven, CT, USA

[10]Yale Univ. School of Medicine, Depts. Of Genetics and Neuroscience, New Haven, CT, USA

[11]VA San Diego Healthcare System, San Diego, CA, USA

[12]Department of Psychiatry, University of California San Diego, La Jolla, CA, USA

*corresponding author, mel41@cam.ac.uk

Contents

- Supplementary Table 1: Genome-wide association study details
- Supplementary Table 2: Code and data availability
- Supplementary Table 3: Statistical comparison of original vs. conditional stratified linkage disequilibrium score regression (s-LDSC models) by *Z*-test
- Supplementary Table 4: Disease-specific T cell acetylation peaks annotated by class of regulatory elements
- Supplementary Figure 1: Enrichment of trans-diagnostic risk at active regulatory elements (active promoters and enhancers) in 88 tissues from the Roadmap epigenomics consortium
- Supplementary Figure 2: Cross-disorder psychiatric risk is enriched in brain and lymphoid immune cell active genomic elements
- Supplementary Figure 3: Enrichment of genetic risk for multiple psychiatric at epigenetically activated sites in adult and fetal brain tissue and immune cells for 8 specific disorders (raw *P*-values)
- Supplementary Figure 4: Enrichment of genetic risk for multiple psychiatric disorders in immune tissues
- Supplementary Figure 5: Psychiatric genetic risk enrichment at active lymphoid enhancers/promoters (BLUEPRINT dataset)
- Supplementary Figure 6: Psychiatric genetic risk enrichment at activation-dependent T cell enhancers/promoters (Soskic immune stimulation dataset)
- Supplementary Figure 7: Soskic stimulated immune cell dataset: overlap of H3K27ac peaks implicated by different disorders
- Supplementary Figure 8: Soskic stimulated immune cell dataset: pathway enrichment for genes nearest to peaks both specific to T cells and overlapped by risk variants
- Supplementary Figure 9: Disease association statistics for variants overlapping T cell specific H3K27ac peaks in the Blueprint and Soskic datasets

Supplementary Table 1 Genome-wide association study details. Genetic variants associated trans-diagnostically with risk for 8 psychiatric disorders and cis-diagnostically with risks for each of 5 specific psychiatric / neurodevelopmental disorders, and 3 positive control disorders. Loci associated with risk were thresholded at $P < 5x10^{-8}$, then distance-based clumping was used to define independently significant loci (see **Methods**).

**Study**	**Number cases**	**Number controls**	**Number of genome-wide independently significant loci**	**Download link**
Cross-disorder psychiatric risk [1]	162,151	276,846	115	https://pgcdata.med.unc.edu/cross_disorder/pgc_cdg2_meta_no23andMe_oct2019_v2.txt.daner.txt.gz
Depression [2]	264,984	581,929	122	dbGaP Study Accession: phs001672.v6.p1
Schizophrenia [3]	36,989	113,075	108	https://pgcdata.med.unc.edu/schizophrenia/ckqny.scz2snpres.gz
Bipolar disorder [4]	20,352	31,358	16	https://www.med.unc.edu/pgc/download-results/ File = daner_PGC_BIP32b_mds7a_0416a
Autism [5]	18,382	27,969	2	https://pgcdata.med.unc.edu/autism_spectrum_disorders/iPSYCH-PGC_ASD_Nov2017.gz
Attention deficit hyperactivity disorder [6]	19,099	34,194	10	https://pgcdata.med.unc.edu/adhd/adhd_eur_jun2017.gz
Body mass index [7]	806,834	NA	1023	https://zenodo.org/record/1251813#.X_iGVS-I1TZ File = bmi.giant-ukbb.meta-analysis.combined.23May2018.txt
Alzheimer's disease [8]	71,880	383,378	25	https://ctg.cncr.nl/documents/p1651/AD_sumstats_Jansenetal_2019sept.txt.gz
Rheumatoid arthritis [9]	14,361	43,923	48	http://plaza.umin.ac.jp/~yokada/datasource/files/GWASMetaResults/RA_GWASmeta_European_v2.txt.gz

Supplementary Table 2 Code and data availability

**Resource**	**Availability**
Code used to perform this analysis and generate the figures in the paper	https://github.com/maryellenlynall/psychimmgen2021 Archived at Zenodo under accession code 7125661 [https://doi.org/10.5281/zenodo.7125661]
Summary statistics	See **Supplementary Table 1**
Roadmap Epigenomics datasets	http://bx.psu.edu/~yuzhang/Roadmap_ideas/trackDb_test.txt
BLUEPRINT datasets	https://www.blueprint-epigenome.eu
Soskic immune stimulation dataset (H3K27ac)	https://www.ebi.ac.uk/ega/studies/EGAS00001002749
IDEAS annotations	http://bx.psu.edu/~yuzhang/Roadmap_ideas/trackDb_test.txt
1000 genomes called against GRCh38	http://ftp.1000genomes.ebi.ac.uk/vol1/ftp/data_collections/1000_genomes_project/release/20190312_biallelic_SNV_and_INDEL/
CHEERS code	https://github.com/trynkaLab/CHEERS
Partitioned LD scores for active regulatory elements in Roadmap tissues	Generated in this analysis; available at https://doi.org/10.5281/zenodo.5153661
GnomAD v2.1.1	https://gnomad.broadinstitute.org

Supplementary Table 3 Statistical comparison of original vs. conditional s-LDSC models by one-sided *Z*-test (see **Methods**), to accompany **Figure 1b** and **Figure 1c**.

	Original model vs. conditional model including fetal male brain (**Fig 1b**)	Original model vs. conditional model including fetal female brain (**Fig 1b**)
Brain Angular Gyrus	z=1.80; p=0.04	z=2.19; p=0.01
Brain Anterior Caudate	z=1.74; p=0.04	z=2.20; p=0.01
Brain Cingulate Gyrus	z=1.75; p=0.04	z=2.19; p=0.01
Brain Germinal Matrix	z=2.61; p=0.005	z=2.92; p=0.002
Brain Hippocampus Middle	z=1.50; p=0.07	z=1.99; p=0.02
Brain Inferior Temporal Lobe	z=1.80; p=0.04	z=2.19; p=0.01
Brain Dorsolateral Prefrontal Cortex	z=1.94; p=0.03	z=2.31; p=0.01
Brain Substantia Nigra	z=1.34; p=0.09	z=1.77; p=0.04
Fetal Brain (female)	z=1.73; p=0.04	NA
Fetal Brain (male)	NA	z=2.77; p=0.003
Immune cell subsets for which trans-risk showed significant enrichment (q < 0.05) in original s-LDSC model	Original model vs. conditional model including all 10 significantly enriched brain regions (**Fig 1c**)	
T cytotoxic naive cells (peripheral blood)	z=0.34; p=0.37	
T helper naive cells (peripheral blood) 2	z=0.20; p=0.42	
T cytotoxic memory cells (peripheral blood)	z=0.21; p=0.42	
T helper 17 cells (PMA-I stimulated)	z=0.19; p=0.42	
T helper cells (PMA-I stimulated)	z=0.19; p=0.42	
T helper memory cells (peripheral blood) 1	z=0.17; p=0.43	
T regulatory cells (peripheral blood)	z=0.20; p=0.42	
T effector/memory (peripheral blood)	z=0.26; p=0.40	
T helper cells (peripheral blood)	z=0.20; p=0.42	
T helper naive cells (peripheral blood) 1	z=0.28; p=0.39	
T helper memory cells (peripheral blood) 2	z=0.18; p=0.43	
T cells (cord blood)	z=0.23; p=0.41	

Supplementary Table 4 Disease-specific T cell acetylation peaks annotated by class of regulatory elements The properties of T cell specific H3K27ac peaks overlapped by cis-diagnostic GWAS risk variants for MDD only, or for schizophrenia only, were compared. Upper table shows the overlap of MDD and schizophrenia T cell specific peaks from the Blueprint dataset, i.e., unstimulated T cells, with regulatory elements defined in unstimulated peripheral blood T cells from the Roadmap dataset. Lower table shows the overlap of MDD and schizophrenia T cell specific peaks from the Soskic dataset, i.e., stimulated T cells, with regulatory elements defined in the Roadmap PMA-stimulated T cells. *P*-values show the results of two-sided X^2 tests (for proportion of peaks overlapping each annotation) or two-sided Mann-Whitney U (MWU) tests (for distance to nearest transcription start site, TSS). Separate chi-squared tests are performed for each annotation because although the annotations themselves are exclusive categories, a given peak can overlap more than one annotation. For each dataset, the FDR column indicates *P*-values corrected for the four tests performed for that dataset (Benjamini-Hochberg correction). There were no significant differences between MDD-only and schizophrenia-only T cell peaks in their overlap with regulatory elements or their distance from the nearest TSS.

**T cell acetylation peaks associated with genetic risk for MDD or schizophrenia: Blueprint dataset (i.e., non-stimulated cells)**					
	**MDD-only peaks**	**Schizophrenia-only peaks**	**Statistic (df)**	***P* value**	**FDR**
Overlap with T cell non-genic enhancers	39%	28%	$X^2(1) = 3.17$	0.07	0.1
Overlap with T cell genic enhancers	38%	25%	$X^2(1) = 4.16$	0.04	0.1
Overlap with T cell promoters	22%	13%	$X^2(1) = 2.27$	0.1	0.2
Distance to nearest TSS (median)	14892 bp	14747 bp	MWU W = 5339.5	0.6	0.6
**T cell acetylation peaks associated with genetic risk for MDD or schizophrenia: Soskic dataset (i.e., stimulated cells)**					
	**MDD-only peaks**	**Schizophrenia-only peaks**	**Statistic (df)**	***P* value**	**FDR**
Overlap with stimulated T cell non-genic enhancers	51%	52%	$X^2(1) = 0.17$	0.7	0.9
Overlap with stimulated T cell genic enhancers	48%	47%	$X^2(1) = 0.44$	0.5	0.9
Overlap with stimulated T cell promoters	33%	33%	$X^2(1) = 0.02$	0.9	0.9
Distance to nearest TSS (median)	11141 bp	9258 bp	MWU W = 5650	0.5	0.9

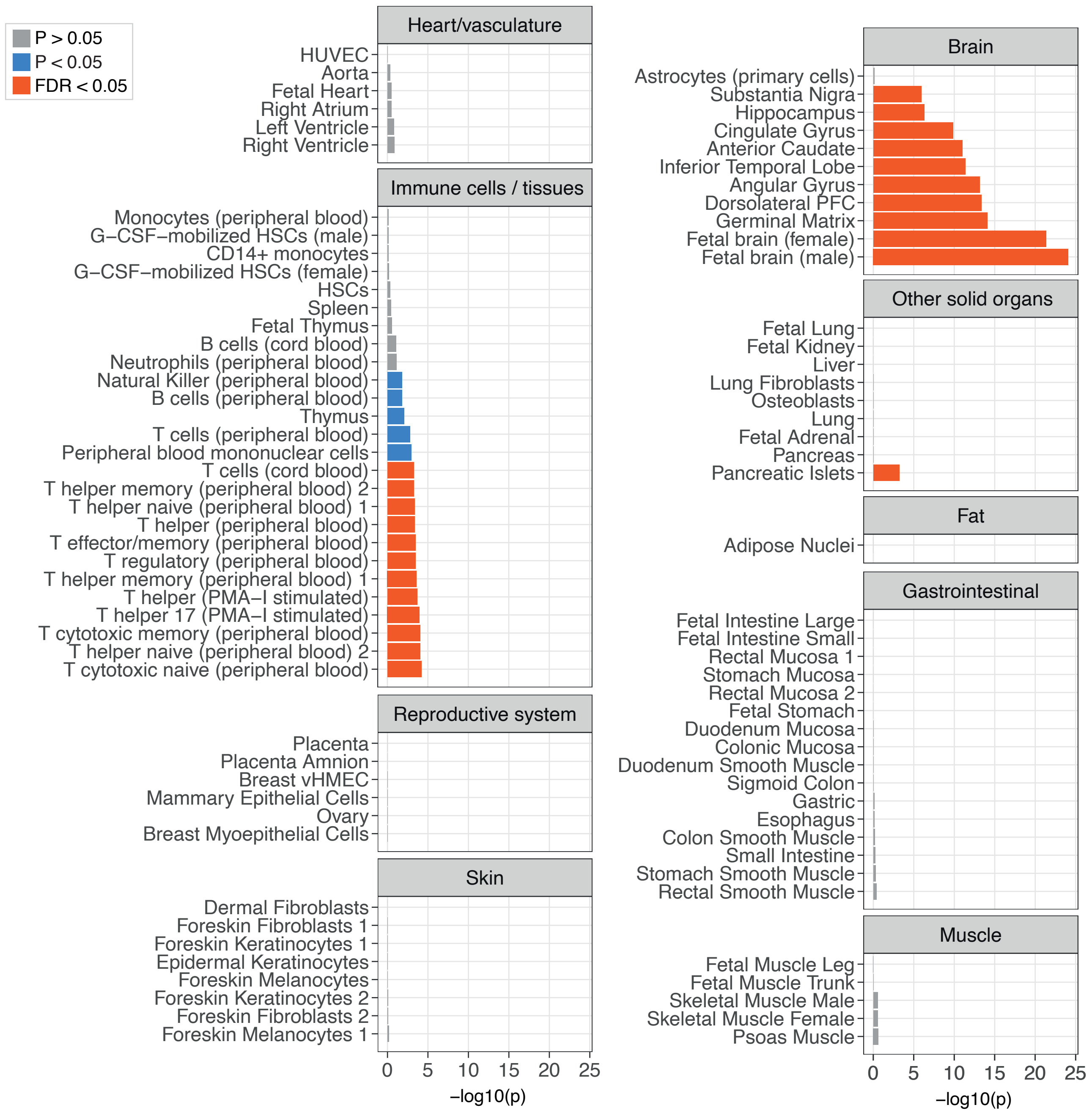

Supplementary Figure 1 Enrichment of trans-diagnostic risk at active regulatory elements (active promoters and enhancers) in 88 tissues from the Roadmap epigenomics consortium *P*-values are shown for the results of stratified linkage disequilibrium score regression (s-LDSC) analysis, taking the union of active elements in a given cell type as the annotation of interest (see **Methods**). The *P*-values from s-LDSC regressions (one-sided tests) were used to test the null hypotheses that risk variants were not co-located with epigenetically activated sites more frequently than expected by chance, at two probability thresholds: $P < 0.05$ (blue); and Benjamini-Hochberg $FDR < 0.05$ (orange), to correct for multiple tests across N=88 tissues. HUVEC, human umbilical vein endothelial cells; vHMEC, variant human mammary epithelial cells; PFC, prefrontal cortex; HSC, hematopoietic stem cell; PMA-I, phorbol-myristate-acetate and ionomycin. A subset of these results is shown in **Figure 1a**. See **Supplementary Table 4** for full statistics.

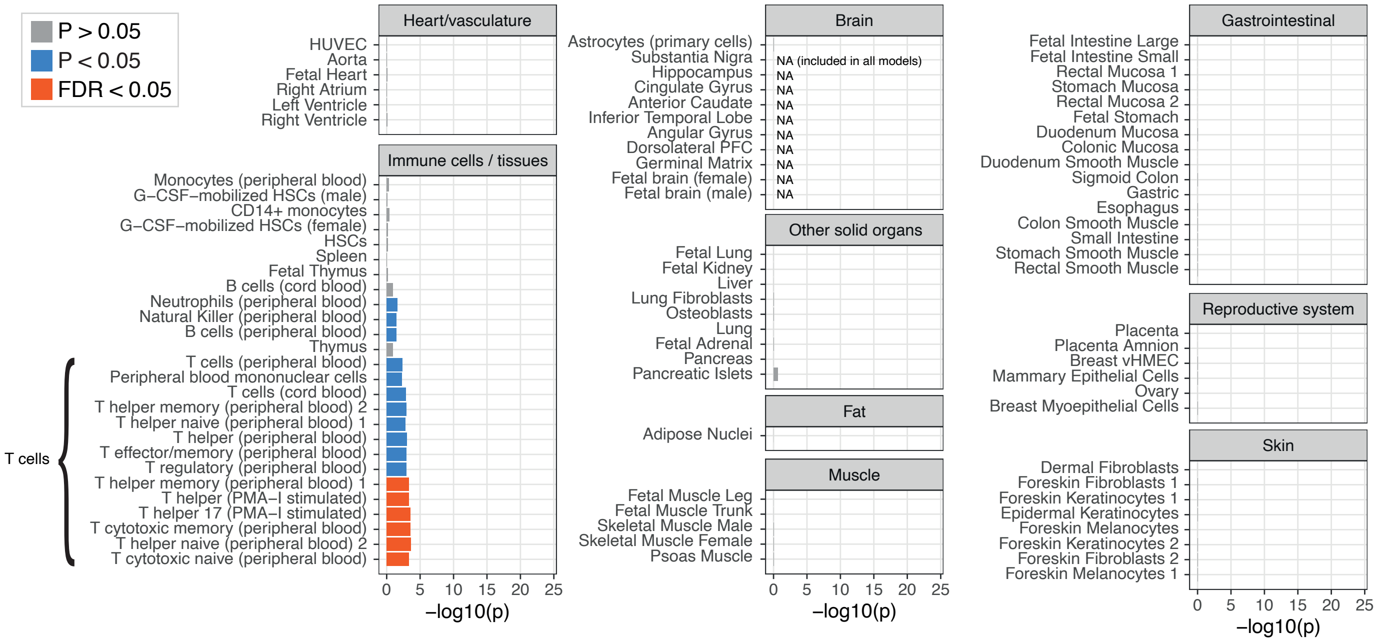

Supplementary Figure 2 Cross-disorder psychiatric risk is enriched in brain and lymphoid immune cell active genomic elements Brain-conditioned analysis: repeat of the analysis in **Figure 1a** and **Supplementary Figure 1**, including the active regulatory annotations for all 10 significantly enriched brain regions as additional terms in the s-LDSC models for all other cell types. The *P*-values from s-LDSC regressions (one-sided tests) were used to test the null hypotheses that risk variants were not co-located with epigenetically activated sites more frequently than expected by chance, at two probability thresholds: *P* < 0.05 (blue); and Benjamini-Hochberg *FDR* < 0.05 (orange), to correct for multiple tests across N=88 tissues. HUVEC, human umbilical vein endothelial cells; vHMEC, variant human mammary epithelial cells; PFC, prefrontal cortex; HSC, hematopoietic stem cell; PMA-I, phorbol-myristate-acetate and ionomycin. A subset of these results is shown in **Figure 1c**.

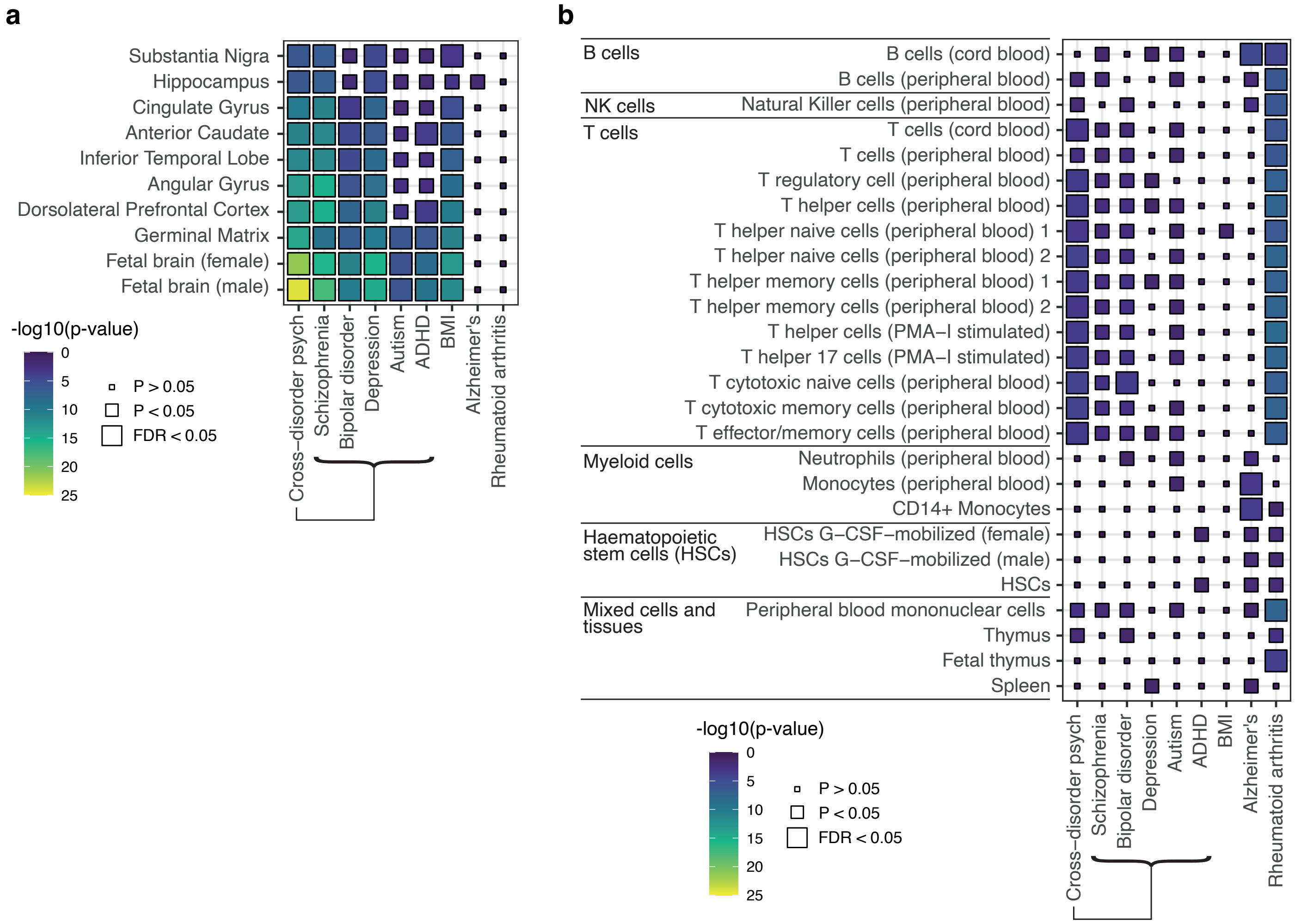

Supplementary Figure 3: Enrichment of genetic risk for multiple psychiatric at epigenetically activated sites in adult and fetal brain tissue and immune cells for 8 specific disorders (raw *P*-values) For each of 5 mental health disorders (schizophrenia, bipolar disorder, major depressive disorder [MDD], autism, and attention deficit-hyperactivity disorder [ADHD]), and for each of 3 positive control disorders (obesity, Alzheimer's disease and rheumatoid arthritis), enrichment of cis-risk variants at active regulatory elements (active promoters and enhancers) was tested in (a) 10 brain tissue samples (3 fetal) and (b) 26 immune cell classes (3 fetal) [10]. *P*-values are shown for the results of stratified linkage disequilibrium score regression (s-LDSC) analysis (one-sided tests), taking the union of active elements in a given cell type as the annotation of interest. Tile size, from large to small, indicates *P*-value thresholds from *FDR* < 0.05 (significant after Benjamini-Hochberg correction for all 88 tissues tested, including those not shown here), through *P* < 0.05 (nominally significant), to *P* ≥ 0.05 (not significant). Full statistics are given in **Supplementary Table 4**. ADHD, attention deficit hyperactivity disorder; BMI, body mass Index.

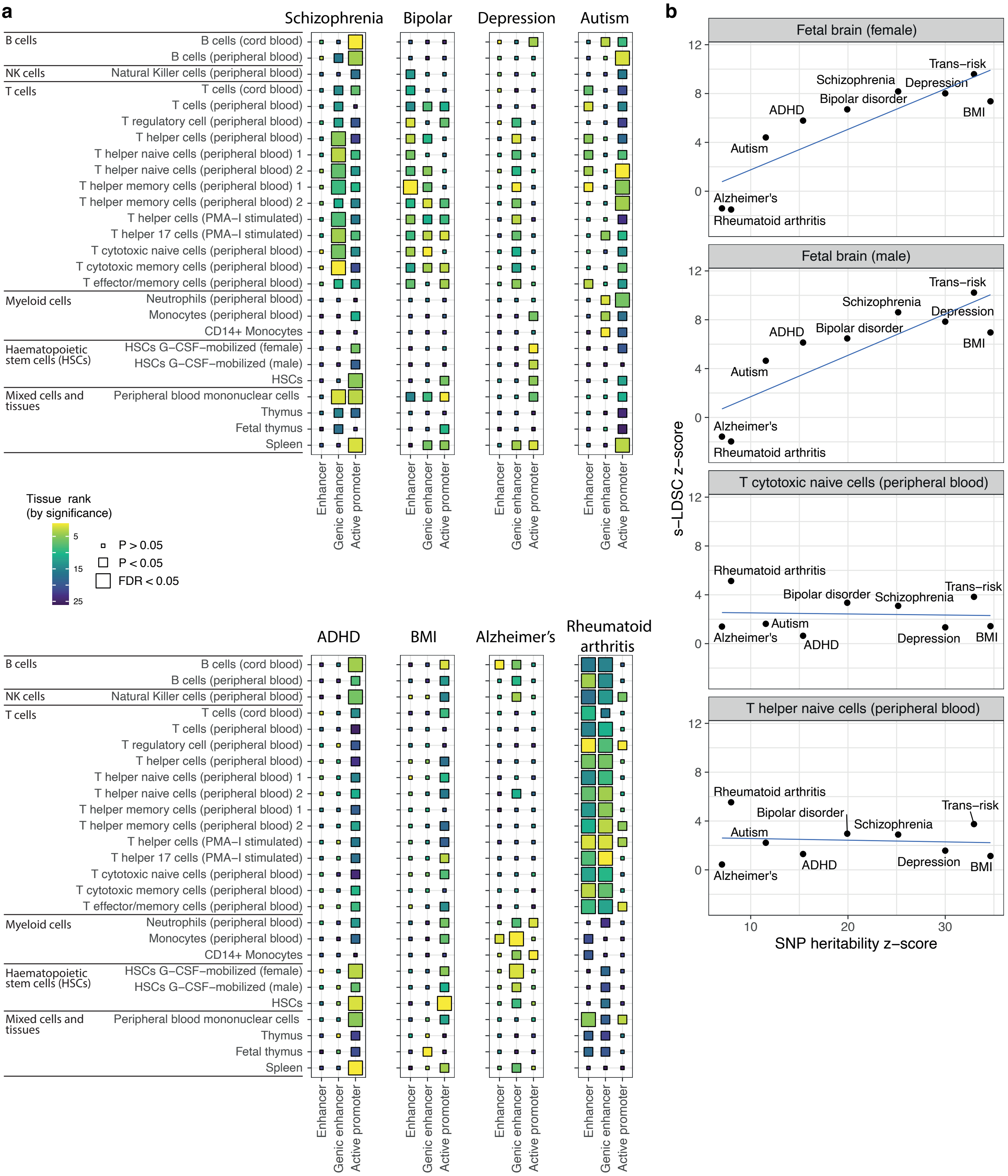

Supplementary Figure 4 Enrichment of genetic risk for multiple psychiatric disorders in immune tissues (a) Enrichment at immune cell enhancers, genic enhancers and active promoters in Roadmap immune tissues. Enrichment at each annotation was calculated using stratified linkage disequilibrium score regression (s-LDSC). The *P*-values from s-LDSC regressions (one-sided tests) indicate the significance of the coefficient for the cell type specific annotations. Tile size indicates significance with FDR correction across all 78 annotations tested. Tile fill indicates

the *P*-value rank (ranking within each annotation across cell types). (b) Correlations between GWAS SNP heritability *Z*-scores and s-LDSC *Z*-scores for the top two brain and immune annotations for trans-risk. Spearman's correlations with heritability z-score are as follows (two-sided test): fetal male brain: Spearman's correlation S(7)=16, $P = 0.005$, $\rho = 0.87$, 95% CI 0.35-1; fetal female brain: S(7)=16, $P = 0.005$, $\rho = 0.87$, 95% CI 0.35-1); cytotoxic T cells: S(7)=120, $P = 1$, $\rho = 0$, 95% CI -0.7-0.7; helper T cells: S(7)=116, $P = 0.9$, $\rho = 0.03$, 95% CI -0.9-0.8).

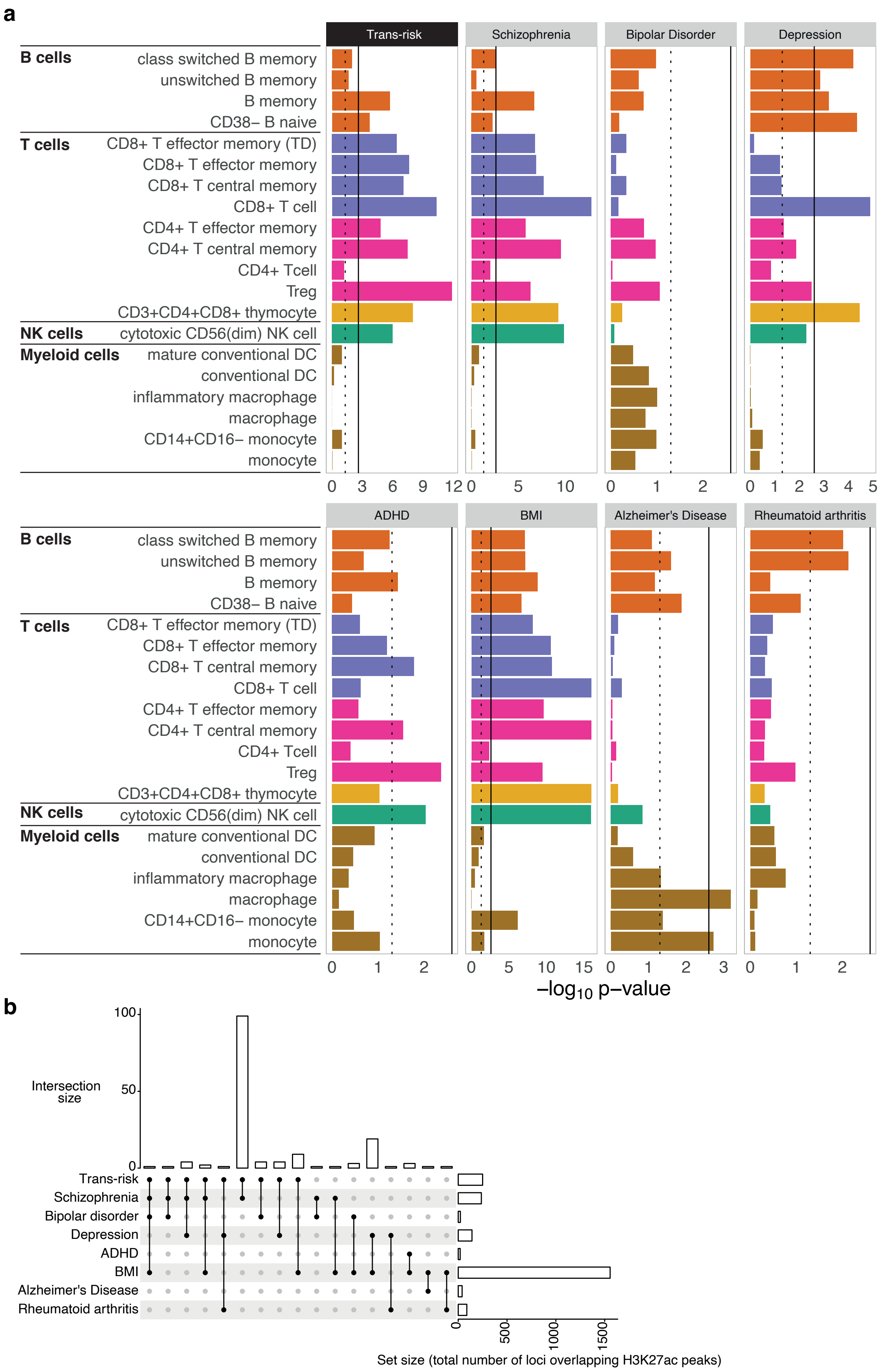
a
Trans-risk
Schizophrenia
Bipolar Disorder
Depression
ADHD
BMI
Alzheimer's Disease
Rheumatoid arthritis
B cells
class switched B memory
unswitched B memory
B memory
CD38– B naive
T cells
CD8+ T effector memory (TD)
CD8+ T effector memory
CD8+ T central memory
CD8+ T cell
CD4+ T effector memory
CD4+ T central memory
CD4+ Tcell
Treg
CD3+CD4+CD8+ thymocyte
NK cells
cytotoxic CD56(dim) NK cell
Myeloid cells
mature conventional DC
conventional DC
inflammatory macrophage
macrophage
CD14+CD16– monocyte
monocyte
–log10 p–value
b
Intersection size
Trans-risk
Schizophrenia
Bipolar disorder
Depression
ADHD
BMI
Alzheimer's Disease
Rheumatoid arthritis
Set size (total number of loci overlapping H3K27ac peaks)

Supplementary Figure 5 Psychiatric genetic risk enrichment at active lymphoid enhancers/promoters (BLUEPRINT dataset) (a) Bar plots show enrichment of genetic risk for each condition at active promoters/enhancers (H3K27ac marks) in unstimulated sorted immune cells. CHEERS was used to detect enrichment of risk loci at cell-type specific H3K27ac peaks by quantifying (for each cell type) the mean cell type specificity score (for that cell type) of peaks overlapping genetic risk variants (see **Methods**). *P*-values are reported from a discrete uniform distribution (one-sided tests). The dotted black line marks the nominal significance threshold, $P < 0.05$; the solid black line marks the Bonferroni-corrected significance threshold, $P_{Bonf} < 0.05$. Note differing x-axis scales. (b) Upset plot for all BLUEPRINT H3K27ac immune peaks overlapped by risk variants for each disorder, showing counts (vertical bars) of shared peaks, compared to total peak number implicated by each disorder (horizontal bars). ADHD, attention deficit hyperactivity disorder; BMI, body mass index.

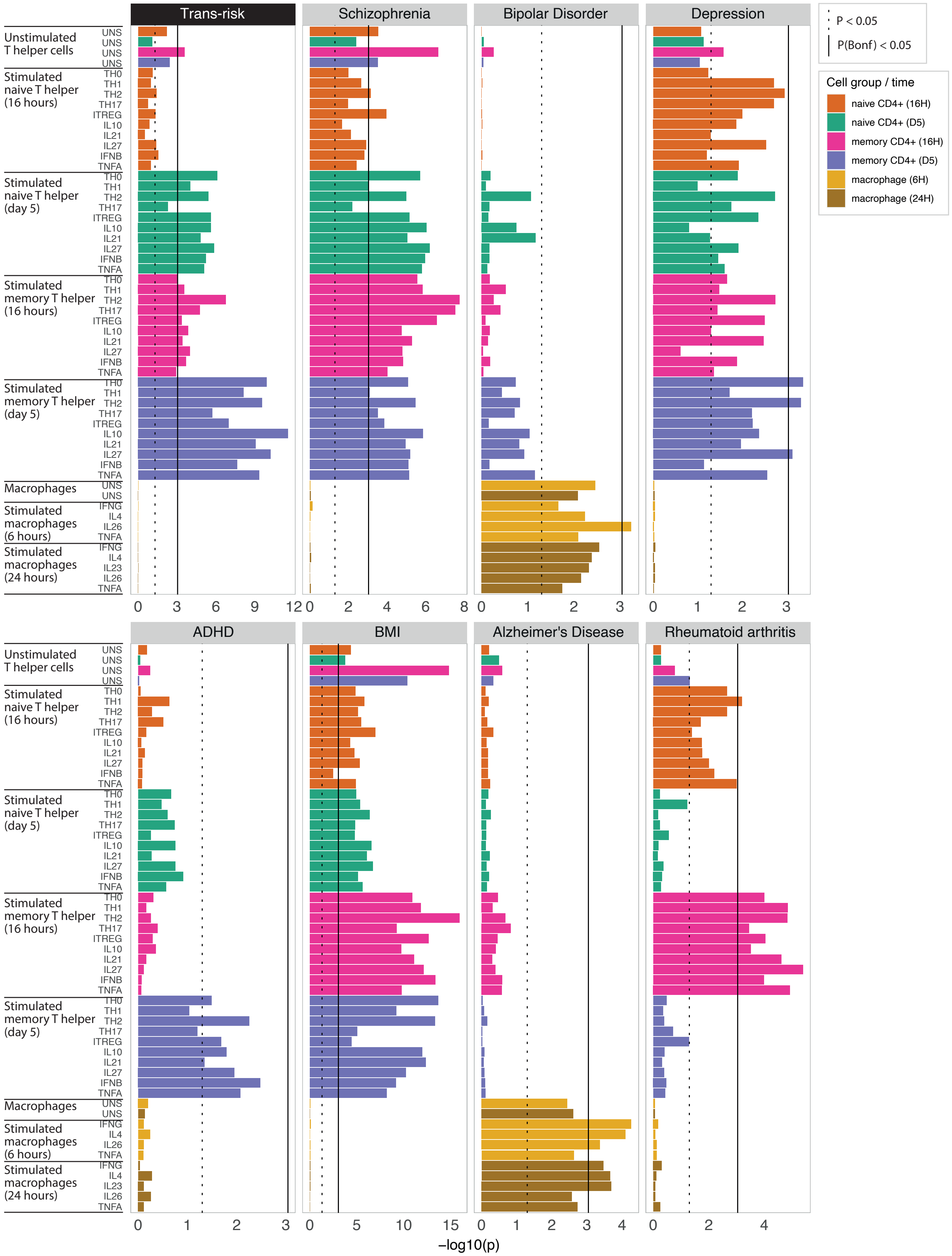

Supplementary Figure 6 Psychiatric genetic risk enrichment at activation-dependent T cell enhancers/promoters (Soskic immune stimulation dataset). Bar plots show enrichment of

genetic risk for each condition at active promoters/enhancers (H3K27ac marks) in unstimulated and *ex vivo* stimulated immune cells. Stimulated cells are sorted macrophages, naïve CD4^{+} (helper) T cells and memory CD4^{+} T cells, assayed at both early and late timepoints (see legend). CHEERS was used to detect enrichment of risk loci at cell-type specific H3K27ac peaks by quantifying, for each cell type, the mean cell type specificity score (for that cell type) of peaks overlapping genetic risk variants (see **Methods**). One-sided *P*-values are reported from a discrete uniform distribution. The dotted black line marks raw $P < 0.05$; the solid black line marks Bonferroni-corrected $P_{Bonf} < 0.05$. Note differing x-axis scales. ADHD, attention deficit hyperactivity disorder; BMI, body mass index.

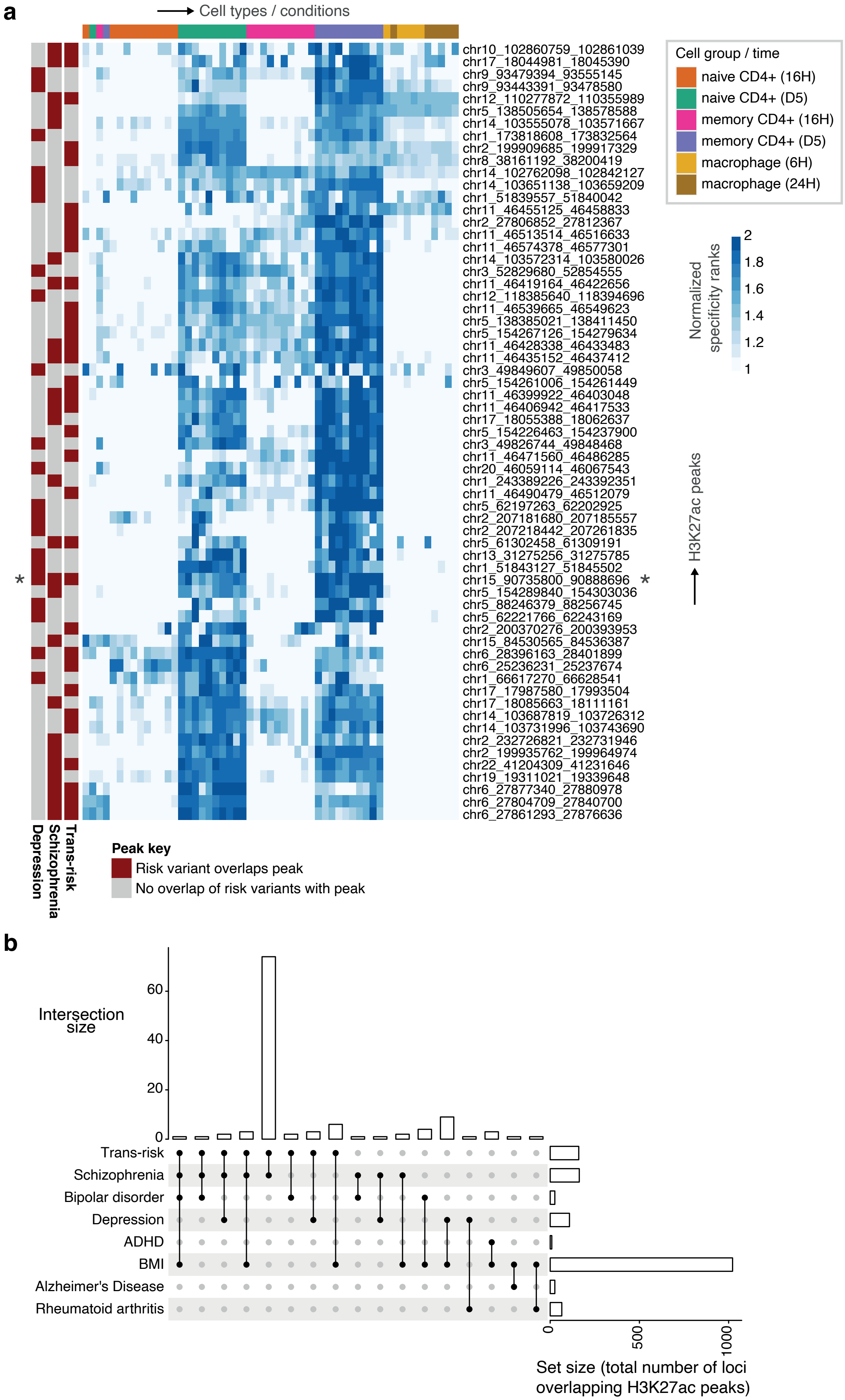
a
Cell types / conditions
Cell group / time
naive CD4+ (16H)
naive CD4+ (D5)
memory CD4+ (16H)
memory CD4+ (D5)
macrophage (6H)
macrophage (24H)
Normalized specificity ranks
2
1.8
1.6
1.4
1.2
1
H3K27ac peaks
chr10_102860759_102861039
chr17_18044981_18045390
chr9_93479394_93555145
chr9_93443391_93478580
chr12_110277872_110355989
chr5_138505654_138578588
chr14_103555078_103571667
chr1_173818608_173832564
chr2_199909685_199917329
chr8_38161192_38200419
chr14_102762098_102842127
chr14_103651138_103659209
chr1_51839557_51840042
chr11_46455125_46458833
chr2_27806852_27812367
chr11_46513514_46516633
chr11_46574378_46577301
chr14_103572314_103580026
chr3_52829680_52854555
chr11_46419164_46422656
chr12_118385640_118394696
chr11_46539665_46549623
chr5_138385021_138411450
chr5_154267126_154279634
chr11_46428338_46433483
chr11_46435152_46437412
chr3_49849607_49850058
chr5_154261006_154261449
chr11_46399922_46403048
chr11_46406942_46417533
chr17_18055388_18062637
chr5_154226463_154237900
chr3_49826744_49848468
chr11_46471560_46486285
chr20_46059114_46067543
chr1_243389226_243392351
chr11_46490479_46512079
chr5_62197263_62202925
chr2_207181680_207185557
chr2_207218442_207261835
chr5_61302458_61309191
chr13_31275256_31275785
chr1_51843127_51845502
chr15_90735800_90888696 *
chr5_154289840_154303036
chr5_88246379_88256745
chr5_62221766_62243169
chr2_200370276_200393953
chr15_84530565_84536387
chr6_28396163_28401899
chr6_25236231_25237674
chr1_66617270_66628541
chr17_17987580_17993504
chr17_18085663_18111161
chr14_103687819_103726312
chr14_103731996_103743690
chr2_232726821_232731946
chr2_199935762_199964974
chr22_41204309_41231646
chr19_19311021_19339648
chr6_27877340_27880978
chr6_27804709_27840700
chr6_27861293_27876636
Depression
Schizophrenia
Trans-risk
Peak key
Risk variant overlaps peak
No overlap of risk variants with peak
b
Intersection size
60
40
20
0
Trans-risk
Schizophrenia
Bipolar disorder
Depression
ADHD
BMI
Alzheimer's Disease
Rheumatoid arthritis
0
500
1000
Set size (total number of loci overlapping H3K27ac peaks)

Supplementary Figure 7 Soskic stimulated immune cell dataset: overlap of H3K27ac peaks implicated by different disorders (a) Heatmap shows the subset of peaks with specificity for late-activated naïve and/or memory $CD4^+$ T cells which are also overlapped by risk variants for either trans-risk, schizophrenia, or major depressive disorder. Each row corresponds to a H3K27ac peak overlapping a risk variant; each column corresponds to a different cytokine-induced cell state (see legend), ordered as in **Figure 4A**. Blue fill shade represents how specific each peak is to each cell state (specificity rank of the peak normalized to the mean specificity rank of all peaks). Row annotations indicate peaks which overlap (dark red) or do not overlap (grey) risk variants for the disorder indicated. Of the late-activation T cell specific peaks, only 1 (starred *) is overlapped by both schizophrenia and depression risk variants. (b) Upset plot for all Soskic dataset H3K27ac immune peaks overlapped by risk variants for each disorder, showing counts (vertical bars) of shared peak overlaps, compared to total number of peaks implicated by each disorder (horizontal bars). ADHD, attention deficit hyperactivity disorder; BMI, body mass index.

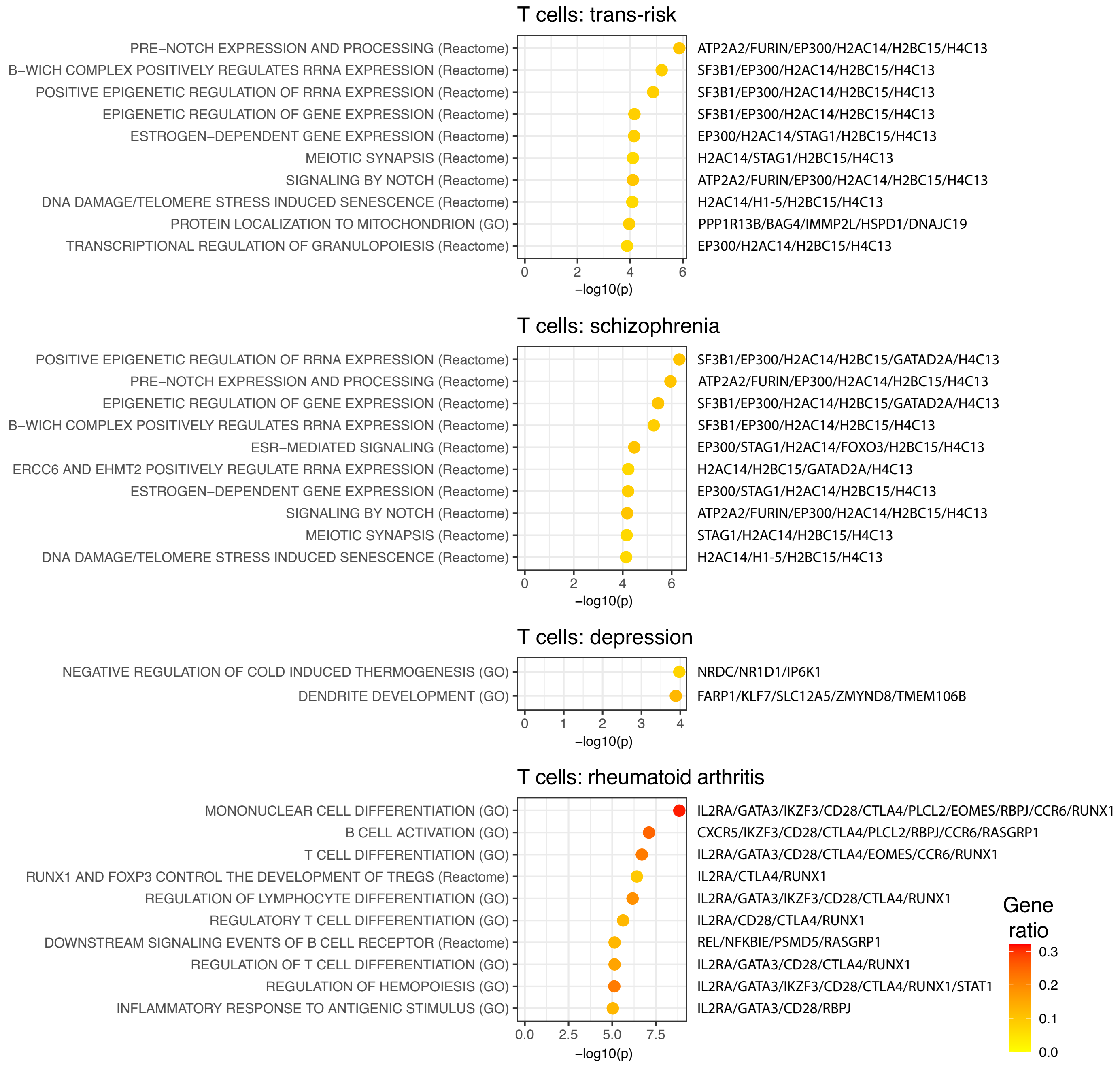

Supplementary Figure 8 Pathway enrichment for genes nearest to T cell acetylation peaks co-located with trans-diagnostic and cis-diagnostic variants. Plots show the most significantly enriched pathways in T cells for each disorder or group of disorders, based on the genes nearest to the T cell-specific H3K27ac peaks co-located with risk variants. *X*-axes show log scale *P* values for enrichment of the pathways (one-sided hypergeometric tests); circle fill shows gene ratio (number of nearest genes in the pathway / total number of test genes); listed genes are those genes in the enriched pathway nearest to the T cell peaks. Plots are shown only for those disorders which showed enrichment of risk variants in T cell subsets. Pathway over-representation analysis was performed using Reactome and GO (Gene Ontology) Biological Process pathways. Only pathways with FDR < 0.05 are shown, with a maximum of 10 pathways shown per disorder or group of disorders.

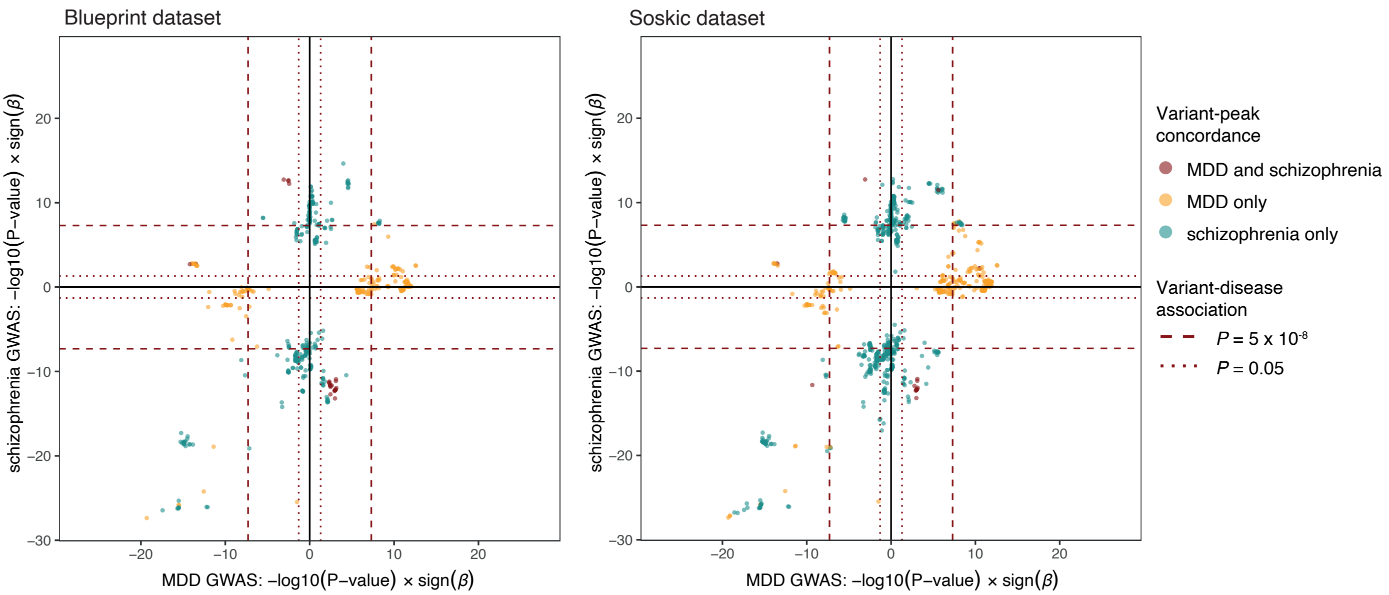

Supplementary Figure 9 Disease association statistics for variants overlapping T cell specific H3K27ac peaks in the Blueprint and Soskic datasets Scatter plots show the cis-diagnostic GWAS statistics independently estimated for MDD (major depressive disorder, x-axis) and schizophrenia (y-axis) at each variant that was co-located with MDD-schizophrenia discordant T cell acetylation peaks (peaks implicated in one but not both disorders). Statistics are shown for the T cell H3K27ac peaks implicated in both the Blueprint (left hand side) and Soskic (right hand side) epigenetic datasets. Axes use the log scale for disease association *P*-values, multiplied by the sign of the beta coefficient, to indicate the direction of effect of the variant on disease risk. Lines show the conventional threshold for genome-wide significance of association for both directions of effect in both disorders ($P = 5 \times 10^{-8}$, dashed lines) and the uncorrected threshold ($P = 0.05$, dotted lines). Many of the variants co-located with discordant T cell acetylation peaks have different signs (negative vs. positive) or strengths of association with the two disorders. For the MDD-schizophrenia discordant histone acetylation peaks, in the Soskic dataset, only 40% of the 649 T cell variant-peak overlaps defined by genome-wide $P < 5 \times 10^{-8}$ were significantly associated with both disorders, with the same sign of association, even at the nominal level of $P < 0.05$; and 24% of the variant-peak overlaps in one disorder had an opposite sign of association with the other disorder. In the Blueprint dataset, only 39% of the 337 T cell variant-peak overlaps defined by genome-wide $P < 5 \times 10^{-8}$ were significantly associated with both disorders, with the same sign of association, even at the nominal level of $P < 0.05$; and 23% of the variant-peak overlaps in one disorder had an opposite sign of association with the other disorder. If histone acetylation at risk variants for MDD and schizophrenia involved the same loci, but a specific variant simply did not reach genome-wide significance for one of the disorders, we would expect the datapoints to cluster along the $y=x$ line in this plot.

Supplementary references

1 Cross-Disorder Group of the Psychiatric Genomics Consortium. Genomic Relationships, Novel Loci, and Pleiotropic Mechanisms across Eight Psychiatric Disorders. *Cell* 179, 1469-1482 e1411, doi:10.1016/j.cell.2019.11.020 (2019).

2 Levey, D. F. *et al.* Bi-ancestral depression GWAS in the Million Veteran Program and meta-analysis in >1.2 million individuals highlight new therapeutic directions. *Nat Neurosci*, doi:10.1038/s41593-021-00860-2 (2021).

3 Schizophrenia Working Group of the Psychiatric Genomics, C. Biological insights from 108 schizophrenia-associated genetic loci. *Nature* 511, 421-427, doi:10.1038/nature13595 (2014).

4 Stahl, E. A. *et al.* Genome-wide association study identifies 30 loci associated with bipolar disorder. *Nat Genet* 51, 793-803, doi:10.1038/s41588-019-0397-8 (2019).

5 Grove, J. *et al.* Identification of common genetic risk variants for autism spectrum disorder. *Nat Genet* 51, 431-444, doi:10.1038/s41588-019-0344-8 (2019).

6 Demontis, D. *et al.* Discovery of the first genome-wide significant risk loci for attention deficit/hyperactivity disorder. *Nat Genet* 51, 63-75, doi:10.1038/s41588-018-0269-7 (2019).

7 Pulit, S. L. *et al.* Meta-analysis of genome-wide association studies for body fat distribution in 694 649 individuals of European ancestry. *Hum Mol Genet* 28, 166-174, doi:10.1093/hmg/ddy327 (2019).

8 Jansen, I. E. *et al.* Genome-wide meta-analysis identifies new loci and functional pathways influencing Alzheimer's disease risk. *Nat Genet* 51, 404-413, doi:10.1038/s41588-018-0311-9 (2019).

9 Okada, Y. *et al.* Genetics of rheumatoid arthritis contributes to biology and drug discovery. *Nature* 506, 376-381, doi:10.1038/nature12873 (2014).

10 Roadmap Epigenomics, C. *et al.* Integrative analysis of 111 reference human epigenomes. *Nature* 518, 317-330, doi:10.1038/nature14248 (2015).
