# Supplementary material for: Genetic variants associated with psychiatric disorders are enriched at epigenetically active sites in lymphoid cells
Source: Nat Commun. 2022 Oct 15;13:6102. doi: 10.1038/s41467-022-33885-7 (PMC9569335; doi:10.1038/s41467-022-33885-7)
Supplement: Supplementary file 3 — Description of Additional Supplementary Files [file 41467_2022_33885_MOESM3_ESM.pdf]

Description of Additional Supplementary Files

File Name: Supplementary Data 1
Description: Statistics for enrichment of genetic risk for psychiatric disorders (trans-risk and cis-risks) in 88 Roadmap tissues. Statistics are shown for the results of stratified linkage disequilibrium score regression (s-LDSC) analyses (one-sided tests), taking the union of active elements in a given cell type / tissue as the annotation of interest. Spreadsheet tabs correspond to results for trans-risk and cis-risk for each disorder. Results correspond to those shown in Figure 1, Figure 2, Supplementary Figure 1 and Supplementary Figure 3. *FDR* indicates adjusted *P*-values following Benjamini-Hochberg correction for all 88 tissues tested. CI_95 indicated the 95% confidence intervals for the enrichment coefficient (effect size). HUVEC, human umbilical vein endothelial cells; vHMEC, variant human mammary epithelial cells; PFC, prefrontal cortex; HSC, hematopoietic stem cell; PMA-I, phorbol-myristate-acetate and ionomycin.

File Name: Supplementary Data 2
Description: T cell genes highlighted by the epigenetic analysis of trans-risk and cis-risk for schizophrenia and depression. T cell-specific peaks in the Soskic immune stimulation dataset which were overlapped by disease risk variants were selected. The genes overlapping those peaks or with transcription start sites nearest to those peaks are reported here. See Supplementary Figure 8 for enrichment analyses corresponding to these results.
