# Supplementary material for: Genetic variants associated with psychiatric disorders are enriched at epigenetically active sites in lymphoid cells
Source: Nat Commun. 2022 Oct 15;13:6102. doi: 10.1038/s41467-022-33885-7 (PMC9569335; doi:10.1038/s41467-022-33885-7)
Supplement: Supplementary file 6 — Reporting Summary [file 41467_2022_33885_MOESM6_ESM.pdf]

Corresponding author(s): Dr Mary-Ellen Lynall

Last updated by author(s): 29/9/22

Reporting Summary

Nature Portfolio wishes to improve the reproducibility of the work that we publish. This form provides structure for consistency and transparency in reporting. For further information on Nature Portfolio policies, see our Editorial Policies and the Editorial Policy Checklist.

Statistics

For all statistical analyses, confirm that the following items are present in the figure legend, table legend, main text, or Methods section.

n/a	Confirmed	
☐	☒	The exact sample size (*n*) for each experimental group/condition, given as a discrete number and unit of measurement
☐	☒	A statement on whether measurements were taken from distinct samples or whether the same sample was measured repeatedly
☐	☒	The statistical test(s) used AND whether they are one- or two-sided<br>*Only common tests should be described solely by name; describe more complex techniques in the Methods section.*
☐	☒	A description of all covariates tested
☐	☒	A description of any assumptions or corrections, such as tests of normality and adjustment for multiple comparisons
☐	☒	A full description of the statistical parameters including central tendency (e.g. means) or other basic estimates (e.g. regression coefficient) AND variation (e.g. standard deviation) or associated estimates of uncertainty (e.g. confidence intervals)
☐	☒	For null hypothesis testing, the test statistic (e.g. *F*, *t*, *r*) with confidence intervals, effect sizes, degrees of freedom and *P* value noted<br>*Give P values as exact values whenever suitable.*
☒	☐	For Bayesian analysis, information on the choice of priors and Markov chain Monte Carlo settings
☒	☐	For hierarchical and complex designs, identification of the appropriate level for tests and full reporting of outcomes
☐	☒	Estimates of effect sizes (e.g. Cohen's *d*, Pearson's *r*), indicating how they were calculated

Our web collection on statistics for biologists contains articles on many of the points above.

Software and code

Policy information about availability of computer code

Data collection	No software was used for data collection.
Data analysis	Custom code for this study provided at https://github.com/maryellenlynall/psychimmgen2021 and is archived at Zenodo under accession code 7125661 [https://doi.org/10.5281/zenodo.7125661]<br>CHEERS v1 https://github.com/TrynkaLab/CHEERS<br>IDEAS annotations: http://bx.psu.edu/~yuzhang/Roadmap_ideas/trackDb_test.txt<br>plink v1.9<br>GnomAD v2.1.1<br>LDSC v1.0.1 with baseline model v1.2 https://github.com/bulik/ldsc

For manuscripts utilizing custom algorithms or software that are central to the research but not yet described in published literature, software must be made available to editors and reviewers. We strongly encourage code deposition in a community repository (e.g. GitHub). See the Nature Portfolio guidelines for submitting code & software for further information.

Data

Policy information about availability of data

All manuscripts must include a data availability statement. This statement should provide the following information, where applicable:

- Accession codes, unique identifiers, or web links for publicly available datasets
- A description of any restrictions on data availability
- For clinical datasets or third party data, please ensure that the statement adheres to our policy

All datasets used for this analysis are publicly available (see Supplementary Table 2). The partitioned LD scores for active regulatory elements in Roadmap tissues

generated in this study have been deposited at Zenodo under accession code 5153661 [https://doi.org/10.5281/zenodo.5153661].

Supplementary Table 2:
Code used to perform this analysis and generate the figures in the paper https://github.com/maryellenlynall/psychimmgen2021
Summary statistics See Supplementary Table 1
Roadmap Epigenomics datasets http://bx.psu.edu/~yuzhang/Roadmap_ideas/trackDb_test.txt
BLUEPRINT datasets https://www.blueprint-epigenome.eu
Soskic immune stimulation dataset (H3K27ac) https://www.ebi.ac.uk/ega/studies/EGAS00001002749
IDEAS annotations http://bx.psu.edu/~yuzhang/Roadmap_ideas/trackDb_test.txt
1000 genomes called against GRCh38 http://ftp.1000genomes.ebi.ac.uk/vol1/ftp/data_collections/1000_genomes_project/release/20190312_biallelic_SNV_and_INDEL/
CHEERS code https://github.com/trynkaLab/CHEERS
Partitioned LD scores for active regulatory elements in Roadmap tissues Generated in this analysis; available at https://doi.org/10.5281/zenodo.5153661
GnomAD v2.1.1 https://gnomad.broadinstitute.org

Field-specific reporting

Please select the one below that is the best fit for your research. If you are not sure, read the appropriate sections before making your selection.

☒ Life sciences ☐ Behavioural & social sciences ☐ Ecological, evolutionary & environmental sciences

For a reference copy of the document with all sections, see nature.com/documents/nr-reporting-summary-flat.pdf

Life sciences study design

All studies must disclose on these points even when the disclosure is negative.

Sample size	The human genetic datasets used are large publicly available meta-analyses of genome wide association studies provided by the psychiatric genomics consortium or other large consortia., in order to maximize our statistical power. Sample sizes for each meta-analysis are provided in Supplementary Table 1.
Data exclusions	The Roadmap dataset was used. Long-term cultured tissues were excluded from analysis as these were not relevant to the hypotheses to be tested.
Replication	Main findings replicated in 3 epigenetic datasets.
Randomization	Not relevant - not an experimental study.
Blinding	Not relevant - not an experimental study.

Reporting for specific materials, systems and methods

We require information from authors about some types of materials, experimental systems and methods used in many studies. Here, indicate whether each material, system or method listed is relevant to your study. If you are not sure if a list item applies to your research, read the appropriate section before selecting a response.

Materials & experimental systems

n/a	Involved in the study	
☒	☐	Antibodies
☒	☐	Eukaryotic cell lines
☒	☐	Palaeontology and archaeology
☒	☐	Animals and other organisms
☒	☐	Human research participants
☒	☐	Clinical data
☒	☐	Dual use research of concern

Methods

n/a	Involved in the study	
☒	☐	ChIP-seq
☒	☐	Flow cytometry
☒	☐	MRI-based neuroimaging
